# Environment, Leptin Sensitivity, and Hypothalamic Plasticity

**DOI:** 10.1155/2013/438072

**Published:** 2013-07-18

**Authors:** Marco Mainardi, Tommaso Pizzorusso, Margherita Maffei

**Affiliations:** ^1^CNR Neuroscience Institute, Via Moruzzi 1, 56124 Pisa, Italy; ^2^Department of Neuroscience, Psychology, Drug Research and Child Health NEUROFARBA, University of Florence, Via San Salvi 12, 50135 Florence, Italy; ^3^Dulbecco Telethon Institute at Endocrinology Unit, University Hospital of Pisa, Via Paradisa 2, 56124 Pisa, Italy; ^4^CNR Institute of Food Sciences, Via Roma 64, 83100 Avellino, Italy

## Abstract

Regulation of feeding behavior has been a crucial step in the interplay between leptin and the arcuate nucleus of the hypothalamus (ARC). On one hand, the basic mechanisms regulating central and peripheral action of leptin are becoming increasingly clear. On the other hand, knowledge on how brain sensitivity to leptin can be modulated is only beginning to accumulate. This point is of paramount importance if one considers that pathologically obese subjects have high levels of plasmatic leptin. A possible strategy for exploring neural plasticity in the ARC is to act on environmental stimuli. This can be achieved with various protocols, namely, physical exercise, high-fat diet, caloric restriction, and environmental enrichment. Use of these protocols can, in turn, be exploited to isolate key molecules with translational potential. In the present review, we summarize present knowledge about the mechanisms of plasticity induced by the environment in the ARC. In addition, we also address the role of leptin in extrahypothalamic plasticity, in order to propose an integrated view of how a single diffusible factor can regulate diverse brain functions.

## 1. Introduction

Food intake is one of the most relevant aspects of metabolic homeostasis. Feeding behavior is the result of the integrated action of peripheral organs and brain. In particular, the arcuate nucleus of the hypothalamus (ARC) has a prominent role in sensing long-term energy stores and in consequently regulating food intake. Indeed, the ARC is positioned around the third ventricle, where the blood-brain barrier is particularly leaky, and is the primary target organ of leptin. In particular, leptin exerts its action by modulating the activity of the two main cell populations of the ARC, NPY, and POMC neurons.

## 2. Overview of Neural Circuits Controlling Response to Leptin

In the ARC, two main neuronal populations of leptin-responsive neurons exist. Their activity has opposite behavioral outcomes and can be identified according to the expression of the neuropeptides proopiomelanocortin (POMC) and *α*-melanocyte-stimulating hormone (*α*-MSH), or Neuropeptide y (NPY) and agouti-related protein (AgRP), respectively [1]. POMC neurons are considered to be the principal cells of the ARC [2]. However, recent work has shown that at least a fraction of POMC cells displays a GABAergic phenotype [3]; the precise role of this population still has to be addressed in detail. On the other hand, NPY cells can be considered as ARC interneurons, since they are GABAergic and synapse onto POMC neurons to modulate their activity [2]. In addition to this local role, however, NPY cells also send projections to nearby hypothalamic nuclei [4].

Both POMC and NPY neurons express the active form of the leptin receptor (ObRb). Leptin has easy access to the ARC, since permeability of the blood-brain barrier around this circumventricular organ is higher than in the rest of the brain. ObRb binding to its ligand activates in both POMC and NPY neurons two main signal transduction pathways: STAT3 and PI3 Kinase/AKT [5]. However, leptin has opposite actions on the two neuronal populations of the ARC. Indeed, activation of leptin signaling results in repression of transcription of the NPY gene, whereas it has an opposite outcome on POMC mRNA synthesis [5]. Moreover, leptin is able to modulate the electrical activity of both NPY and POMC cells, by acting on L-type calcium channels [6], whereas it also operates on TRPC nonselective cation channels on the latter cell type alone [7]. This results in decrease of Ca^2+^ influx in NPY neurons, which are thus hyperpolarized, whereas an opposite action is exerted on POMC neurons, which become depolarized. The net effect of leptin on the ARC is to favor POMC neuron activity, in order to promote activity of anorexigenic pathways at the expense of orexigenic pathways, with the final action of repressing food intake and increasing energy expenditure.

ARC neurons in turn send projections to nearby hypothalamic nuclei, in addition to subcortical areas (preoptic area) and to the nucleus of tractus solitarius of the vagus nerve [4]. Thus, leptin sensing by the ARC is able to interact with and modulate the activity of several brain regions involved in metabolism and feeding behavior.

The first experiments aimed at elucidating the role of the mediobasal hypothalamus were based on chemical ablation; this coarse approach leads to the development of hyperphagia [8] and contributes to highlight the importance of this brain area in feeding behavior regulation. More recently, genetic approaches have permitted a more precise ablation of selected neural populations of the ARC. Gene ablation of POMC neurons causes hyperphagia [9]. On the other hand, the outcome of AgRP/NPY neurons manipulation is more variable, ranging from the expected hypophagia if performed in adulthood [10] to paradoxical hyperphagia if performed during immediate postnatal development [11]. This age-dependent effect may reflect behavioral adaptation to precocious loss of AgRP neurons, in addition to suggesting that other pathways, possibly outside of the ARC, can compensate for loss of these neuronal types. However, the preeminence of NPY and POMC neurons in regulating feeding behavior should not be questioned. Indeed, elegant experiments using optogenetic activation of AgRP neurons have clearly shown that activation of this neuronal population leads to immediate voracious feeding [12]. Further experiments using a similar approach on POMC neurons still have to be performed. Recent evidence shows that targeted pharmacological activation of POMC neurons has an effect on food intake and body weight only after 3 days of treatment, wher as no effect is detected acutely within 24 hours from treatment [13]. Basing on these results, we predict that POMC neuron stimulation would result in the repression of food intake only if performed chronically for several days, highlighting a subtler role of these cells in repressing food intake, with a reduction in feeding characterized by a slower kinetic compared to the immediate effect produced by AgRP neuron stimulation [12].

On the other hand, optogenetics can be exploited also for inhibiting the activation of NPY and POMC neurons, by means of halorhodopsin expression [14]. Compared to gene ablation, this approach has the advantage of preserving the neuronal population under investigation, thus, allowing to test the functionality of the circuit without substantially altering it.

## 3. Leptin and Its Peripheral Effects

Leptin derives from the Greek term lepthos (thin) and is coded by the *ob* gene. The *ob/ob* mice, harboring a point mutation in this gene, are diabetic, hyperphagic, infertile, and show other abnormalities including a less efficient immune system, high bone mass [15], and impaired thermogenesis [16, 17]. When treated with the recombinant hormone, their food intake and body weight are normalized to that of the lean controls and most of the previously mentioned alterations are completely rescued [18–21]. Similar observations were reported for the few human obese subjects carrying a homozygous mutation in the leptin gene that exhibited a dramatic phenotype and greatly benefited from recombinant leptin therapy [22].

Leptin is mainly expressed by the white adipose tissue (WAT) [23], and long before its molecular identification its presence and nature (a hormone) had been postulated by Doug Coleman in the 1960s, based on parabiosis experiments that utilized *ob/ob* and wild type mice [24]. During these experiments *ob/ob* mice lost weight implying that a regulator of body weight, which they naturally missed, was provided by the blood flow of WT mice. 

In those years, body weight regulation was modeled by Hervey as a typical homeostatic mechanism in which a controller (already hypothesized to reside in the hypothalamus) was informed by the status of the energy stores through an afferent signal able to sense them. In turn the controller, through an efferent signal, was able to control the energy stores and the afferent signal levels in a feedback loop, typical of the endocrine mechanisms [25]. Leptin cloning, followed one year later by the identification of its receptor (OB-R) [26], gave a hard support to this hypothesis. Leptin filled perfectly the requirements of the afferent signal: (i) it was produced by the WAT, the largest energy store in the body, (ii) its expression was directly related to fat mass [27, 28], (iii) it was a bioactive molecule [19] able to reduce body weight, and finally (iv) the active form of its receptor (OB-Rb) was present in the ARC [29]. Concerning point (ii), the upstream signals linking increase in adiposity to induction in leptin expression in WAT are still a matter of investigation: it is known that insulin, glucocorticoids, and the tumor necrosis factor alpha (TNF-*α*) [30, 31] participate to leptin regulation, but evidence so far accumulated does not account for the fine modulation of this hormone.

Leptin discovery was key to disclose a huge amount of knowledge in the field of body weight homeostasis and clarified which molecular pathways concur to determine the fine tuning of satiety and appetite in the brain. However, some aspects of the Hervey model regarding the efferent signals still need to be tested. Until recently leptin-brain relationship has in fact been considered a one-way dialogue with no feedback on the periphery, if we exclude the indirect effects on WAT caused by energy unbalance. 

Given the clear physiological properties and behavioral outcomes of ARC neurons, the process of metabolic homeostasis regulation appears quite straightforward, with appetite repression and energy expenditure being linearly regulated by plasma leptin concentration. However, obese patients typically display high leptin levels, which happens in the absence of any leptin receptor mutation [32]. This raises the possibility that the pathological phenotype is the result of cerebral leptin insensitivity, possibly as a result of wrong set-point hypothalamic circuits to the levels of this hormone. Thus, a crucial issue is to understand if and how leptin sensitivity can be regulated by acting on hypothalamic circuits.

## 4. Neural Plasticity Induced by Environmental Stimuli in the Arcuate Nucleus of the Hypothalamus

Neural plasticity is the process by which neural circuits adapt their response to variations in stimuli coming from the environment. Concerning the hypothalamus, plasticity was first studied as a result of changes in the internal environment, namely, blood concentration of estrogen. It was first shown that GABAergic synapses on ARC neurons are negatively regulated by estradiol [33]. Subsequent work has also demonstrated that synapses formed on dendritic spines, which are mainly excitatory, are sensitive to the estrous cycle [34].

A growing body of the literature addresses the impact on neural plasticity of genetic manipulation [35]. On the other hand and despite the importance of this topic, surprisingly few works address the issue of understanding how the environment affects neural plasticity. A better knowledge of this process is instrumental in finding the key molecules that mediate the beneficial effects of environment on metabolism, with the ultimate goal of designing effective therapies. One of the purposes of the present review is to highlight how recent findings by our and other groups have shed new light on this challenging topic suggesting a fascinating scenario in which the environment, acting as a plasticizing agent on the brain, is able to modulate leptin production and action in the periphery.

During immediate postnatal development, leptin has been shown to regulate correct development of ARC projections to the paraventricular nucleus of the hypothalamus (PVH). Indeed, leptin-deficient *ob/ob* mice display sparser projections to the paraventricular nucleus of the hypothalamus compared to wild-type mice [36]. On the other hand, leptin treatment of *ob/ob* mice is able to normalize the density of these projections, but only if treatment is performed during immediate postnatal life, whereas it is ineffective in the adult [36]. This has led to hypothesizing the existence of a “critical period” for proper development of hypothalamic circuits, in close analogy to what has been shown for cortical sensory systems [37].

In addition, leptin is able to modulate the firing pattern of ARC neurons. Indeed, leptin-deficient *ob/ob* mice display increased inhibitory currents and decreased excitatory synapses on POMC neurons, whereas NPY neurons receive more excitatory synapses. Replacement of leptin is able to normalize these alterations, demonstrating that adult hypothalamic circuits display a plastic potential [38].

On the other hand, one of the most interesting problems is to understand how hypothalamic plasticity can be modulated by environmental stimuli, such as the life style or food intake. To this regard, the main experimental models are (i) physical exercise by running wheel activity; (ii) high-fat diet (HFD) exposure; (iii) caloric restriction. Physical exercise protocols aim at increasing movement activity of the animal; this is usually achieved by placing a running wheel in the cage, with constant or intermittent access [39]. The number of animals that are housed in the same cage is variable, ranging from a few units to a single individual. It should be noted, however, that this may represent a confounding factor for result interpretation, since a low amount of social interaction (i.e., low number of animals in a cage) has been shown to affect food intake [40]. HFD is achieved by changing the standard chow diet for laboratory rodents with special formulas containing a higher amount of fat and carbohydrates [41, 42]. HFD ultimately leads to pathological obesity and type II diabetes and is a valid model for simulating the interplay between excessive caloric intake, metabolic syndrome, and brain [43]. On the other hand, various protocols have been set up for studying the effect of a reduction in caloric intake on the brain. The simplest protocol is caloric restriction that is based on reducing the total daily intake of food to values that span from 70% to 90% of normal intake [44]. A further development is represented by intermittent fasting that consists of a one day on-one day off food availability schedule. Since the animals usually experience a rebound feeding the day after fasting, the total caloric balance, also in this case, is usually reduced to 70–90% of normal intake [45, 46].

More recently, environmental enrichment (EE) has been added to this list [47]. Compared to the simple physical exercise protocol, EE adds cognitive, sensory, and social stimulations by means of rearing larger groups of animals (up to ten, compared to the usual three to five) in wider cages with several objects to explore that are frequently changed [48, 49]. A summary of these protocols is presented in Table 1.

## 5. Molecular Mediators of Hypothalamic Plasticity

Transition to HFD induces an adaptation in ARC circuits which is mediated by a transient increase in the expression of the extracellular matrix component polysialic acid (PSA), whose role in promoting neural plasticity is well known in the cortex [50]. The increase in polysialic acid synthesis correlates with a change in the firing pattern of POMC neurons, which display a higher excitatory activity, possibly to compensate the excess nutrient intake with an inhibition of feeding [51]. Of note, the role of extracellular matrix in regulating adaptation to dietary change was also demonstrated using mice deficient for the tissue inhibitor of metalloproteinase-2 (TIMP-2). TIMP-2 knockouts display higher weight gain and hyperleptinaemia after transition to HFD [52]. However, these compensatory reactions will eventually be ineffective, since HFD will result invariably in obesity and loss of leptin sensitivity. On the other hand, enhancing motor activity with forced physical exercise has been demonstrated to produce lasting improvements in the status of rats with diet-induced obesity (DIO) fed a high-energy diet. Indeed, a brief period of physical exercise reduces body weight and enhances the effects of leptin without requiring a switch to standard diet; moreover, these benefits are maintained even after the end of the exercising period. At the hypothalamic level, this correlates with increased STAT3 phosphorylation, thus pointing to increased leptin sensitivity [42]. Analogous benefits was observed when wild-type mice were subjected to EE since birth. Indeed, they showed a reduction in plasma leptin levels, despite no significant differences in food intake or body weight compared to controls. Strikingly, this pointed to an environmentally induced reprogramming of the set-point for hypothalamic response to the anorexigenic signal transmitted by leptin. This was accompanied by an enhancement in glucose tolerance and food intake repression in response to leptin injection and by an increase in STAT3 phosphorylation in the ARC. In addition, excitatory and inhibitory synapses on the soma of POMC and NPY neurons were quantified to look for explicit signs of neural plasticity. It was found that EE results in an increase in the excitatory tone on POMC neurons and in opposite changes on NPY neurons. Notably, in comparison to physical exercise alone, rearrangement of hypothalamic synapses were observed only in EE mice, whereas increased leptin sensitivity and STAT3 phosphorylation were common to both conditions [47]. It is worth noting that, if EE is terminated, not all these changes are maintained. Indeed, the increased STAT3 phosphorylation in response to leptin is no more observed, whereas an imprint on ARC synapses can still be observed [47]. It is tempting to speculate that this imprint will help in reinstating higher STAT3 phosphorylation in the ARC, after transitioning again to the EE protocol. Moreover, further work would be required to investigate whether the precocious EE experience can also induce resistance to diet-induced obesity.

An important aspect of studies on experience-induced plasticity is the search for the key molecular mediators. In the case of EE, we have shown that enriched animals displayed an increased synthesis of brain-derived neurotrophic factor (BDNF) [47]. Interestingly, this molecule is the ideal bridge between control of feeding behavior and plasticity. Firstly, BDNF-deficient mice are obese [53]. Projections from POMC neurons of the ARC act on the ventromedial hypothalamic nucleus (VMH) to induce synthesis of BDNF via activation of the melanocortin-4 receptor (MC4R) [54]. BDNF in turn is anterogradely transported to synaptic sites, where it is released in an activity-dependent manner [55]. One of the effects of synaptically released BDNF is a facilitation in consolidation of neural circuits, since this neurotrophin is required for the establishment of long-term potentiation (LTP) [56]. However, the precise role of BDNF in repressing food intake still has to be completely elucidated [57].

On the other hand, BDNF had initially been shown to be a master controller of plasticity. BDNF-deficient mice display cognitive impairments and reduced long-term potentiation in the hippocampus [58, 59]. In addition, manipulation of BDNF levels also modulates cortical plasticity [60]. Strikingly, increased BDNF levels are observed as a result of EE, both during the immediate postnatal development and adulthood [61, 62]. 

In addition, recent work has uncovered a previously unknown and beneficial role of the increase of BDNF caused by exposure to EE. In mice inoculated with B16 melanoma cells or in the MC38 colon cancer model, EE ameliorated the pathological phenotype by reducing tumor size [63]. Of note, a decrease in serum leptin level was observed in those experiments, as also reported by us [47]. Strikingly, hypothalamic overexpression of BDNF *per se* was able to replicate the effect of EE on cancer outcome [63]. Moreover, the same results could not be obtained by exposing mice to running wheel activity alone. In the same line of findings is our observation that EE, but not physical exercise, was able to induce synaptic plasticity [47]. Another interesting finding by Cao and colleagues [63] is that the beneficial effects of hypothalamic BDNF are due to activation of adrenergic *β* receptors, since administration of the *β*-blocker propranolol abolishes the decrease in serum leptin associated with EE. Taken together, these results add functional value to the direct polysynaptic projection from the ARC to adipose tissue that has been described anatomically [64] and lead to hypothesizing that the feedback from the brain to WAT can consist of sympathetic fibers.

Another interesting aspect of the effects of EE on the leptin-hypothalamus-adipose tissue axis is the induction of WAT transdifferentiation. Indeed, the appearance of multiloculated adipose cells, characterized by a gene expression profile resembling that of brown adipose tissue (BAT) cells, has been described in EE mice [65] and put in correlation with resistance to diet-induced obesity. Even if the results should be interpreted cautiously, since the perirenal adipose tissue contains *per se *a fraction of brown adipose tissue (Maffei M and Barone I, unpublished observation), these “brite” cells can be responsible for the peripheral changes leading to improved metabolic homeostasis in animals undergoing EE. Again, these effects appear downstream of sympathetic output to the adipose tissue driven by hypothalamic activation [65].

But what translates the beneficial effects of a period of enhanced physical, cognitive, and social stimulations into lasting changes of neural circuits? A first hint comes from the experiments showing rapid modulation of ARC synaptic currents by leptin [66]. However, these seminal experiments only showed the acute effect of leptin on neural activity. Our group has shown that EE induces robust synaptic plasticity in the ARC, namely, through a global reduction in inhibition and an opposite change of excitation [47]. We have analyzed more in detail ARC synaptology in mice raised in EE since birth and found that POMC neurons receive more excitatory inputs, whereas more inhibitory synapses impinge on NPY neurons [47]. These findings indicate that (i) modulation of excitation/inhibition ratio is related to the metabolic changes observed in EE and (ii) the increase in leptin sensitivity that occurs in mice raised in EE since birth could be explained by an increase in the excitatory input on POMC neurons. Of note, plasticity in the ARC appears to be regulated according to the same mechanisms observed in the cerebral cortex [62, 67]. Thus, modulation of the excitation/inhibition ratio seems to be a common motif in brain plasticity.

A further factor to be taken into account is that the ARC has been shown to be one of those few sites where neurogenesis takes place also in adulthood [68]. Moreover, suppression of neurogenesis has been observed in the ARC of mice with diet-induced obesity [41]. Of note, EE induces an increase of adult neurogenesis in the hippocampus [69], whereas the ARC has not been yet investigated to this regard. Although still to be fully demonstrated, we could then build a hypothetical scenario in analogy to what is observed in the hippocampus: neurogenesis in the ARC may act as a further mechanism to sense environmental stimulation and translates it into a sort of “metabolic” memory.

In this context, ARC neurogenesis can also be seen as an important source of neurons to be integrated with newly formed networks and can collaborate with synaptic rearrangements to the reprogramming of leptin sensitivity observed in EE.

## 6. Leptin and Nonhypothalamic Plasticity 

Obesity has also been linked to the induction of cognitive deficits [70], but the underlying mediators are still poorly understood. A growing body of evidence is now pointing to leptin as a regulator of plasticity also outside of hypothalamic and metabolism-related circuits. In fact, expression of leptin receptor isoforms is observed in various parts of the brain, including the hippocampus and cortex [71].

Induction of hyperleptinaemia in rats results in impairment in hippocampal synaptic plasticity, as assessed with induction of long-term potentiation by stimulation of the Schaffer collateral pathway. This deficit is reversed by lowering blood leptin levels by mild caloric restriction [72]. In addition, fasting induces activation of the ERK pathway in the hypothalamus, and *ob/ob *mice are deficient in this response [73]. ERK is one of the key transducers of plasticity-inducing stimuli in the cortex [74, 75]. Further work will be required to assess whether ERK is also sensitive to leptin action outside the hypothalamus. Of note, caloric restriction *per se* has been shown to be a powerful inducer of neural plasticity, up to the point of restoring visual cortical plasticity in adult rats [76]. In this paper, leptin levels were not measured, but it is highly likely that caloric restriction resulted in their decrease, as already shown [77]. Moreover, EE has been shown to restore cortical plasticity in adult rats [62] in addition to decreasing plasmatic leptin levels and increasing leptin sensitivity [47]. This suggests that interfering with leptin action on cortical circuits can be a valuable strategy to restore plasticity. Animal findings can be put in correlation with analogous results in humans, showing that three months of caloric restriction cause an improved cognitive performance [78].

On the other hand, leptin supplementation also can exert a positive effect on plasticity and cognitive performance. Indeed, leptin supplementation has been shown to restore proper cognitive development in a pediatric patient with a mutated leptin gene [79]. Accordingly, hippocampal long-term potentiation and performance in the Morris Water Maze test are impaired in *db/db* mice and in the hyperleptinaemic Zucker rat strain [80]. Moreover, leptin increases hippocampal neurogenesis [81].

 Thus, it is tempting to speculate that also at the extrahypothalamic level, lower circulating leptin associated to higher leptin sensitivity can be relevant for adult plasticity, whereas in immediate postnatal life adequate levels of this hormone are required to support correct development of neural circuits.

## 7. Conclusions and Future Perspectives

Increasing experimental evidence is showing that leptin is not only a hormone but also a molecular link between metabolism, neural plasticity, and cognition. Considering that the ARC is the primary sensor of leptin, it also appears to be a possible orchestrator of the polyvalent effects of leptin, also if one takes into account its widespread projections. On the other hand, the wide expression of ObRb in extrahypothalamic sites calls for a search of direct effects of leptin.

Experiments performed using modulation of environmental inputs, either by EE, physical exercise, or variation of caloric intake, point to a crucial role of BDNF, excitatory tone, extracellular matrix, and neurogenesis in neural plasticity associated with variations in leptin levels. Notably, these parameters have long been known as mediators of hippocampal and cortical plasticity [82, 83], pointing to the existence of common principles regulating adaptation of neural circuits to variations in experience throughout the brain. Moreover, all these variables have been shown to be regulated by EE, in addition to the reduction in leptin levels. On the other hand, efferent signals originating in the brain to regulate activity of WAT (the main leptin-producing site) are beginning to emerge, with remarkable transdifferentiation effect on the adipocyte that can be switched to a “brite” phenotype in certain conditions. 

We believe that future experimental efforts should be aimed at establishing a clear hierarchy between all factors mentioned, in order to build a coherent picture of the leptin-brain interaction. This will be an instrumental step for isolating the master controller(s) of hypothalamic and extrahypothalamic plasticity, with the ultimate goal of designing effective therapies for metabolic syndromes.

## Figures and Tables

**Table 1:** Summary of the main protocols for studying the interactions between environment, leptin, and neural plasticity, with their main features and effects on the ARC. The effects of the various rearing protocols on leptin levels, STAT3 phosphorylation (pSTAT3) in the ARC, and neural plasticity are described as compared to the standard rearing condition (first row).

Protocol	Animals per cage	Running wheel	Objects	Food	Leptin	pSTAT3 in the ARC	Neural plasticity in the ARC
Standard condition	3 to 5	No	No	Standard chow diet, *ad libitum *	Control value	Control value	Control value
Physical exercise	1 to 3	Yes	No	Standard chow diet, *ad libitum *	↓	↑	Not observed on synaptic puncta. No data available for other markers
Diet-induced obesity (DIO)	3 to 5	No	No	High-fat, high-carbohydrate diet, *ad libitum *	↑	↓	Extracellular Matrix Rearrangements
Caloric restriction	3 to 5	No	No	Standard chow diet, 70% to 90% of normal caloric intake, sometimes coupled to intermittent fasting	↓	No data available	No data available
Environmental enrichment	6 to 10	Yes	Yes	Standard chow diet, *ad libitum *	↓	↑	Modulation of excitation to inhibition ratio
